# A comparative analysis of colour preferences in temperate and tropical social bees

**DOI:** 10.1007/s00114-017-1531-z

**Published:** 2018-01-02

**Authors:** G. S. Balamurali, Elizabeth Nicholls, Hema Somanathan, Natalie Hempel de Ibarra

**Affiliations:** 1 School of Biology, Indian Institute of Science Education and Research, Thiruvananthapuram, India; 2 Centre for Research in Animal Behaviour, Department of Psychology, University of Exeter, Exeter, EX4 4QG UK; 3 School of Life Sciences, University of Sussex, Brighton, Falmer, BN1 9QG UK

**Keywords:** Pollination, Sensory ecology, Foraging decisions, Learning and memory, Sensory bias

## Abstract

The spontaneous occurrence of colour preferences without learning has been demonstrated in several insect species; however, the underlying mechanisms are still not understood. Here, we use a comparative approach to investigate spontaneous and learned colour preferences in foraging bees of two tropical and one temperate species. We hypothesised that tropical bees utilise different sets of plants and therefore might differ in their spontaneous colour preferences. We tested colour-naive bees and foragers from colonies that had been enclosed in large flight cages for a long time. Bees were shortly trained with triplets of neutral, UV-grey stimuli placed randomly at eight locations on a black training disk to induce foraging motivation. During unrewarded tests, the bees’ responses to eight colours were video-recorded. Bees explored all colours and displayed an overall preference for colours dominated by long or short wavelengths, rather than a single colour stimulus. Naive *Apis cerana* and *Bombus terrestris* showed similar choices. Both inspected long-wavelength stimuli more than short-wavelength stimuli, whilst responses of the tropical stingless bee *Tetragonula iridipennis* differed, suggesting that resource partitioning could be a determinant of spontaneous colour preferences. Reward on an unsaturated yellow colour shifted the bees’ preference curves as predicted, which is in line with previous findings that brief colour experience overrides the expression of spontaneous preferences. We conclude that rather than determining foraging behaviour in inflexible ways, spontaneous colour preferences vary depending on experimental settings and reflect potential biases in mechanisms of learning and decision-making in pollinating insects.

**Electronic supplementary material:**

The online version of this article (10.1007/s00114-017-1531-z) contains supplementary material, which is available to authorized users.

## Introduction

The question of how foraging insect pollinators find flowers has intrigued naturalists and scientists for a long time. It has been commonly proposed that insects possess innate colour preferences which help them to find the best-rewarding flowers (e.g., Giurfa et al. 1995; Lunau and Maier 1995). Innate colour preferences thus could form part of the ‘search image’, which reflects co-evolutionary adaptations between floral signals and the sensory-neural capacity of pollinators (Menzel 1985). Given that over evolutionary time, many pollinating insects have become fully or strongly dependent on floral rewards, the adaptive value of such innate preferences for colour and other floral features seems quite obvious. However, the mechanistic basis and functional consequences of these preferences remain poorly understood. By definition, in order to be adaptive innate colour preferences would need to be sufficiently hard-wired (also referred to as genetically determined, or formerly as instinctive (Tinbergen 1951)), in other words expressed regardless of differences in developmental conditions and rearing environment. However, this means that if insects are to learn by experience, which we know they are capable of, these preferences must subsequently somehow be inactivated or suppressed when insects learn colours (Menzel 1985; Giurfa et al. 1995; Gumbert 2000).

Colour choices are easily transformed with experience or specific learned colour associations leading to long-lasting colour memories that determine colour choices and generalisation patterns. The simplest explanation thus is that innate preferences are quickly erased as soon as the insect acquires its first colour associations or experiences (Heinrich et al. 1977; Menzel 1985; Giurfa et al. 1995). An alternative view is that spontaneous colour preferences remain unchanged throughout a foragers’ life and continuously influence decisions whenever the animal encounters new situations or interacts with a variety of flowers (Lunau and Maier 1995; Gumbert 2000; Brito et al. 2015). Both views, in one way or another, imply a close mechanistic and functional relationship between the expression of innate colour preferences and the acquisition and recall of colour memories in the individual forager. The mechanisms underlying this relationship are unknown, but usually it is assumed that controlling the environment and development of the insect with respect to their exposure to colour prior to testing is sufficient to evidence innateness of preferences. However, the applied experimental manipulations are likely to affect the development of the brain and behaviour of an individual in some form, and it remains unclear to what extent the test choices shown in experiments reflect innate preferences that would affect the forager’s behaviour under natural conditions. We therefore refer to experimentally measured responses and preference patterns of colour-naive animals as *spontaneous colour preferences*.

Little is yet known about when and why spontaneous colour preferences vary between pollinator species, and whether any variations can be linked to their specialisations on different flower types, both in specialist plant-pollinator relationships or in generalist bees through resource partitioning in a pollinator community. Also, variations might occur if floral communities differ strongly amongst habitats of macroecological regions, such as the tropics or temperate regions. Testing pollinators in a comparative approach, with similar methods and stimuli (e.g. Kandori et al. 2009; Kandori and Yamaki 2012), can be useful for gaining further insights into the adaptiveness and mechanisms underlying spontaneous colour preferences.

We compared spontaneous colour responses in foragers of three generalist species of bees, the Eastern honeybee *Apis cerana*, stingless bee *Tetragonula iridipennis* (formerly *Trigona iridipennis*), and the bumblebee *Bombus terrestris*. The spectral sensitivities of bee photoreceptors are highly conserved, equally spaced, and moderately overlapping across the visual range, imparting bees with a ‘general- purpose’ colour vision system with excellent capabilities for detecting and discriminating colours in the UV and visible range of the spectrum (Kelber 2006; Peitsch et al. 1992; Vorobyev and Menzel 1999; for a review, see Hempel de Ibarra et al. 2014). We can therefore exclude the possibility that any variation would be due to major differences between the visual systems of different bee species. The comparison of tropical and temperate bees is of interest to the general question of how varied spontaneous colour preferences are. For instance, if spontaneous colour preferences reflect evolutionary adaptations to the colour and reward properties of the flowers that bees pollinate, one could expect that the colour preferences of tropical bees might differ when compared to those of temperate bees. Flowering plants in temperate and tropical plant communities and habitats experience distinct biotic and abiotic selection pressures on flowering phenology, abundance, spatial distributions, and floral signals, and may well present substantial divergence in signalling strategies to attract different suites of pollinators (Roubik 1992; Endress 1996; Johnson 2006). In addition, we investigated whether exposure to a rewarded pale colour stimulus might already provide sufficient colour experience to induce changes to the response patterns. To control the bees’ experience with colour, we either reared naive individuals (*A. cerana*, *B. terrestris*) or enclosed hives for several weeks (*A. cerana*). In the case of *T. iridipennis*, we were able to enclose the hives for 3 months in order to allow for a turnover from experienced to naive foragers.

## Methods

Experiments with bees from colonies of *Apis cerana* and *Tetragonula iridipennis* Moure (formerly also referred to as *Trigona iridipennis*) (Rasmussen and Cameron 2007; Michener 2013) were conducted in South India, Kerala, on the IISER Thiruvananthapuram campus. Colonies and flight cages were located outdoor on a roof terrace. A pilot experiment was conducted to observe the behaviour of freely foraging *Apis cerana* bees with our stimulus disc (Fig. S1) and response to our training parameters.

**Fig. 1:**
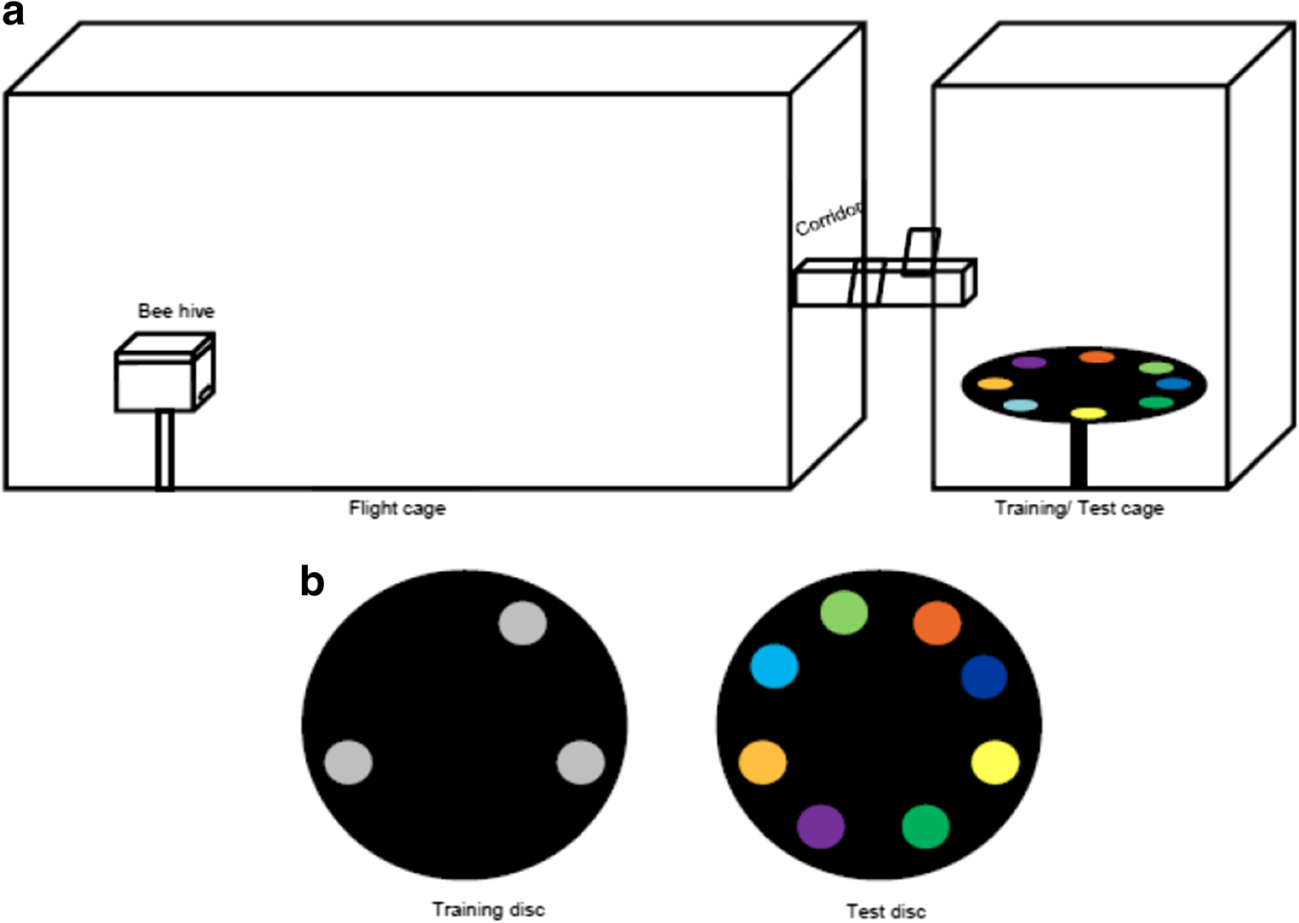
Experimental methods. **a** Flight cage and test cage used for training and testing naïve or enclosed foragers of *A. cerana* and *T .iridipennis*. **b** Training disc showing the triplet of UV-grey stimuli. The position and distances between stimuli were changed in each trial across eight positions. In the test, a test disc was presented to the bees with the coloured stimuli. The order of the stimuli on the test disc was varied across different bees

To obtain colour-naive foragers, single-cohort colonies of *A. cerana* were prepared with newly emerged bees from brood incubated at 30 °C. The colony was placed in a small, 90-cm^3^ flight cage for 1 week in the lab (12/12 light-dark cycle), so that bees could get accustomed to the introduced queen. During this period, the colony was provided with a sucrose-protein bee feed (Neopol, Germany) and water inside the colony box. The hive was then moved into a large outdoor flight cage covered with mesh (3 m × 2.4 m × 1.8 m, Fig. 1a). Freshly emerged bees were added daily to the nucleus colony for up to 2 weeks. During experiments, bees were trained and tested in an adjacent smaller cage (1.2 m × 1.2 m × 1.8 m), under natural, open-sky illumination. The cages were connected to each other by a Plexiglas corridor with controllable doors through which bees were trained to fly to the test cage (Fig. 1a).In the large flight, cage bees were offered 30% (*w*/*w*) sucrose solution in a UV-transparent plastic feeder with black base and mixed-species, bee-collected pollen (Werner-Seip, Germany, dry and freshly ground), that was placed inside a dark box to prevent colour learning. Experiments were conducted from February to April 2013.

In addition to the single-cohort colonies, we also enclosed mature colonies of *A. cerana* for extended periods of time, conducting experiments with foragers after 2, 6, 8, and 9 weeks of enclosure. We assumed that previously formed colour associations in experienced foragers could weaken or decay after some period of time without colour reinforcement. In addition, new naïve foragers would start foraging and older bees could die, as *Apis cerana* has a life span of 4–5 weeks as adult; to compare, in *Apis mellifera* bees reach an age of 4–8 weeks (Dyer and Seeley 1991; Rueppell et al. 2007). Enclosed foragers of *A. cerana* were tested during January to May 2012.

Another group of important tropical pollinators are stingless bees. *Tetragonula iridipennis* is an abundant stingless bee found throughout the South Asian tropics, and it sometimes can be seen to visit similar flowers as *Apis cerana* (H. Somanathan, personal observations). However, given its much smaller body size, it is likely that both species partition their floral resources, and thus might differ in their spontaneous preferences. Since it is not possible to rear naive foragers in *T. iridipennis* without destroying the colony, we enclosed queen-right mature colonies for 3 months (February–April 2013). Experiments were conducted between May and June 2013 in the outdoor flight cages under natural daylight illumination. It is reasonable to assume that after such long time, old foragers were replaced by younger naive bees, given that the life span of *T. iridipennis* foragers has been estimated to be 45 days (Devanesan et al. 2003).

We chose bumblebees as temperate species, because its spontaneous colour preferences have been previously studied, and because suitably small colonies of Western honeybees are difficult to successfully maintain in outdoor flight nets over sufficient time to conduct these experiments and to obtain sufficient numbers of motivated foragers (N. Hempel de Ibarra, personal observations). *Bombus terrestris audax* were purchased from Koppert UK, and experiments were performed outdoor on the Streatham campus of the University of Exeter, UK. A single, queen-right colony, containing workers with no experience of foraging, was housed in a wooden box with a Plexiglas exit box used to release larger-sized foragers individually. The colony was placed in one corner of a large mesh flight cage (4 m × 3 m × 2 m), under natural illumination. Experiments were carried out during September 2012 and from July to August 2013.

### Experimental procedures

Pilot experiments were carried out during April and May 2011 in India with free-flying *A. cerana* foragers to formalise the experimental protocol. In these pilot experiments, free-flying, experienced foragers of *A. cerana* were trained and tested in the open, under natural daylight. Bees were trained to an achromatic stimulus (UV-grey, *n* = 7), and two unsaturated chromatic stimuli (pale blue, *n* = 16; pale yellow, *n* = 7) and their choices in the tests were recorded (Fig. S1).

#### Training phase (*A. cerana*, *T. iridipennis*)

Bees were trained to feed from a transparent feeder with a black base containing 30% sugar solution, which was placed near the hive entrance. The distance of the feeder was gradually increased to motivate the bees to fly towards the test cage. They were trained to fly through a transparent plastic corridor into the test cage to get to become accustomed to the horizontal 80 cm diameter plywood disc covered with black chart paper, on which during a short training period achromatic training stimuli (UV-grey discs, 8 cm in diameter) or chromatic stimuli (pale yellow discs, 8 cm in diameter) were presented (Fig. 1b). The disc had eight positions at which a training stimulus could be placed, three of which were randomly selected per trial. Sugar solution was provided in 0.2 μl micropipette tips in the centre of each stimulus. Bees were marked with water-soluble acrylic paints on the thorax and/or abdomen and individually trained to visit training stimuli during five foraging bouts. The position and grouping of the training stimuli was changed between each bout to preclude the development of positional bias. The stimuli and the micropipette tips were also changed to prevent potentially negative effects of any scent markings during training. Since stingless bees are much smaller in body size, the disc and stimuli were scaled down to half the size i.e. a 40-cm-diameter disc with training stimuli of 4 cm diameter each.

#### Training phase (*B. terrestris*)

Individually released foragers were trained with sucrose solution presented just on the black base of the plastic feeder, to locate the black training disc by slowly increasing the distance to the nest exit over a few steps. Afterwards, they were exposed to either three UV-grey or three pale yellow discs, as described above.

#### Test phase

In a single unrewarded test, the marked bees were presented with a black test disc containing eight coloured test stimuli (diameter 8 cm; Fig. 1b). The test disc for the stingless bees was 40 cm in diameter, displaying eight test stimuli of 4 cm diameter each. In order to control for any potential positional bias, the disc was rotated approximately once every minute, when the bee had flown upwards and away from the test disc, and the positions of the stimuli were changed randomly between bees. The test stimuli were presented for 3 min, and the choice behaviour of bees was recorded in AVHCD format at 25 frames/second using a Sony HDR-160 camcorder. After testing, each bee was removed to avoid pseudo-replication.

### Colour stimuli

**Fig. 2:**
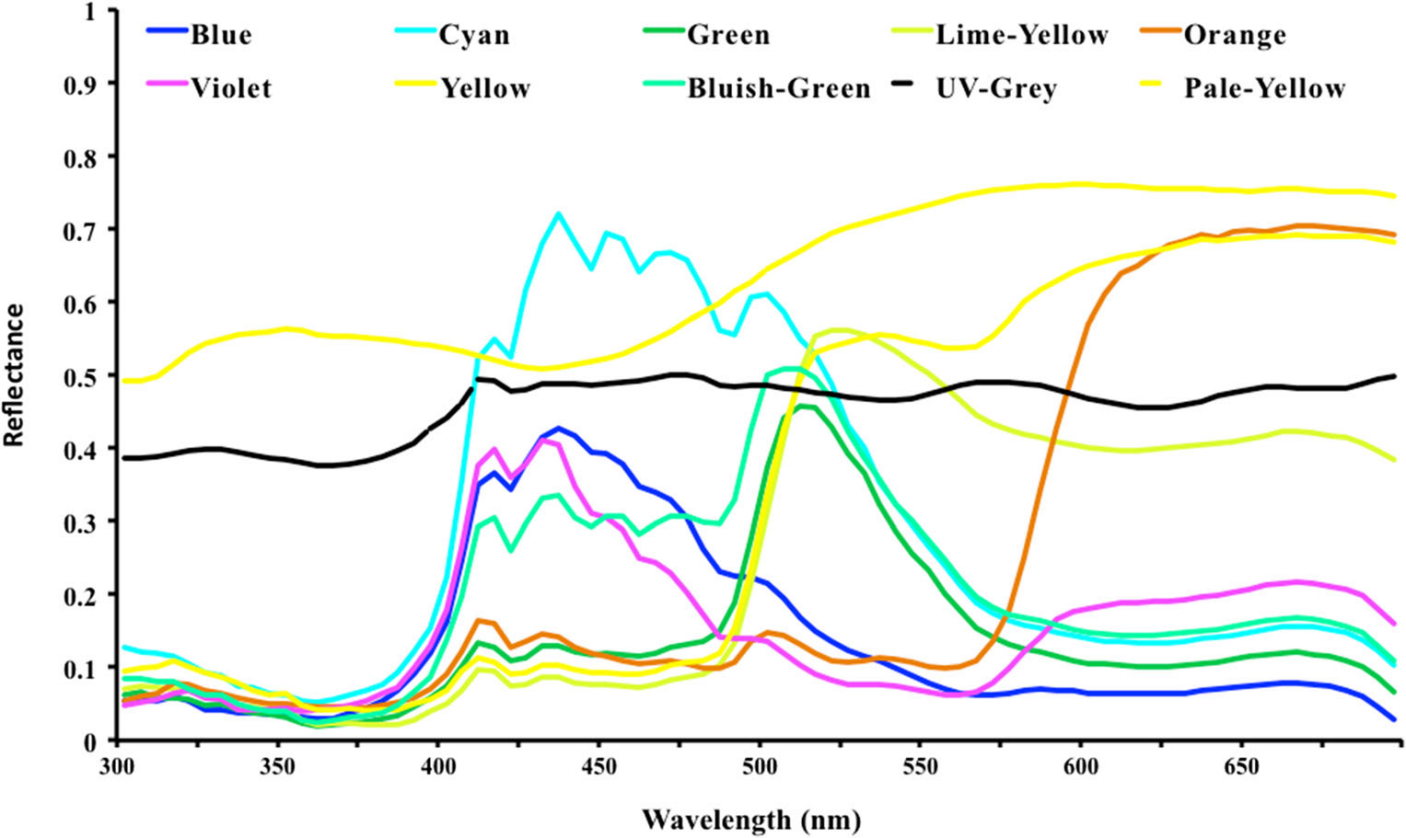
Spectral reflectances of the stimuli used in training and tests. Colour stimuli were designed in Adobe Photoshop (RGB for orange 255/100/0; green 110/187/73; bluish-green 0/255/180; violet 201/1/201; cyan 5/204/246; blue 0/0/255; yellow 246/232/5; lime-yellow 219/244/8)

Adobe Photoshop CS5 was used to create the coloured test stimuli, which were printed on white printer paper (JK Copier, JK Paper Ltd., India) with an Epson Stylus TX121 printer. The UV-grey (50% UV reflecting grey) was purchased as chart paper (brand unknown, all stimuli came from the same purchased batch). The pale yellow training stimulus was cut out from unsaturated yellowish printer paper (Bond, Bilt Ltd., India). Reflectances of stimuli were measured with a spectrophotometer (Maya 2000, Ocean Optics, USA) using a reflectance probe connected to a pulsed xenon light source (PX-2, Ocean Optics, USA) (Fig. 2). The spectra of test colours have strong peaks and will be therefore colourful to bees, whilst the pale yellow colour has a shallower peak, since it is a much less saturated colour. The UV-grey curve is nearly flat as expected for an achromatic stimulus (e.g. Vorobyev et al. 1999).

### Data analysis

The behaviour of the bees during tests was analysed frame-by-frame from video recordings. A choice was recorded when the bee approached a stimulus, scoring it as either an approach with landing or an approach without landing. In an approach without landing the bee would spend some time flying around and above the stimulus before leaving the area and making a new choice.

Randomization tests were used given the data was not normally distributed. The power of randomisation tests can be higher for various effect sizes than that of traditionally applied non-parametric tests (Adams and Anthony 1996), and they have been frequently used for the analysis of behavioural data. The tests were conducted in R (V.3.2.3) using custom code. Initially, we determined for each group of bees their respective treatment sum of squares (*ss*_*t*_) for the measured variable (approaches of each bee to the colours). The data were then randomised 5000 times for each group by shuffling the choices made by a bee. This retained the inter-individual variation in each randomly generated dataset. The corresponding treatment *ss*_*t*_ was calculated in every iteration. The experimental *ss*_*t*_ for each group was then compared to its corresponding frequency distribution of the randomised *ss*_*t*_ to infer whether it fell within a 95% confidence range of randomly generated data, and to calculate a *p* value as the proportion of randomised *ss*_*t*_ that were larger than the experimental *ss*_*t*_.

The test colours were classed as either short-wavelength or long-wavelength stimuli, according to the peak of their reflection spectrum. Tests for equal proportions were conducted in *R* to determine if bees preferred short-wavelength or long-wavelength stimuli.

## Results

### Colour-naive foragers

**Fig. 3:**
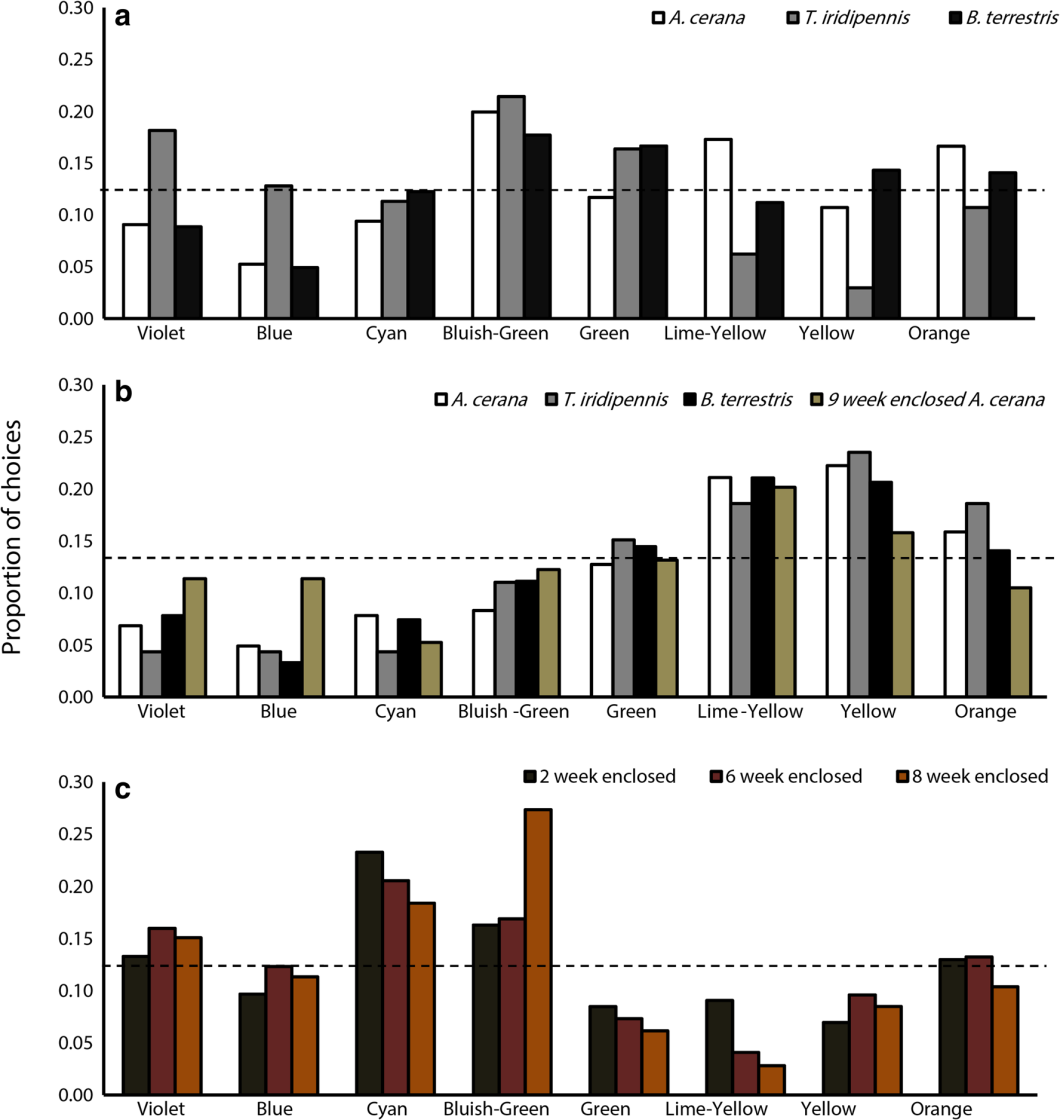
Approaches to the test stimuli in unrewarded tests by **a** enclosed *A. cerana* bees shortly pre-trained to UV-grey stimuli, **b** naive *A. cerana*, *B. terrestris*, and *T. iridipennis* after short training to UV-grey stimuli, and **c** naive and enclosed bees shortly pre-trained to pale yellow stimuli

**Fig. 4:**
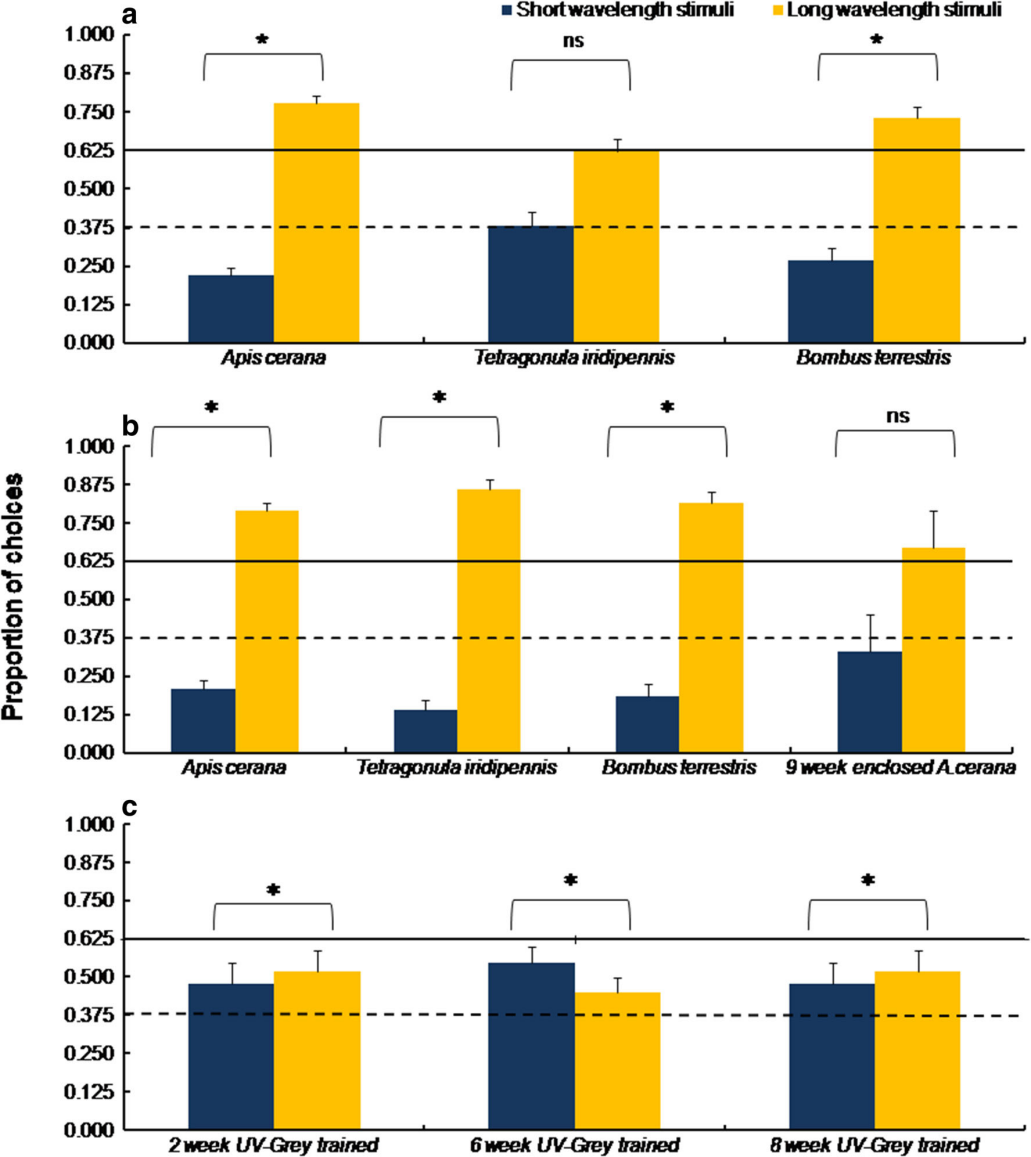
Comparing colour preferences between short- or long-wavelength stimuli. Choices are shown for **a** naive foragers shortly pre-trained to neutral UV-grey, **b** naive and enclosed foragers after short training to pale yellow, and **c** enclosed *A. cerana* foragers pre-trained to UV-grey stimuli. Error bars show standard error; asterisk (*) depicts significance *p* < 0.05

*Apis cerana* foragers shortly pre-trained to the achromatic UV-grey stimuli (*n* = 30 bees) were mostly chose bluish-green and lime-yellow stimuli in the test (*N* = 607 choices; *ss*_*t*_ = 121.49, *p* < 0.01; Fig. 3a). Overall, bees chose long-wavelength stimuli more when choices to stimuli categorised as either short or long wavelength were compared (*χ*^2^ = 9.6, *df* = 1, *p <* 0.01; Fig. 4a). Another group of naive *A. cerana* that were rewarded with sucrose on pale yellow stimuli prior to testing (*n* = 29) approached the yellow stimulus most (*N* = 611 choices; *ss*_*t*_ = 397.72, *p* < 0.01; Fig. 3b), and bees showed a strong overall preference for long-wavelength stimuli (*χ*^2^ = 10.92, *df* = 1, *p* < 0.001; Fig. 4b).

Colour naive *T. iridipennis* foragers (*n* = 20) showed non-random approaches to test colours after training (*N* = 336 choices; *ss*_*t*_ = 147.40, *p* < 0.01, Fig. 3a), exhibiting preferences for the violet and bluish-green stimuli. However, there were no significant differences when choices were categorised to short-wavelength and long-wavelength stimuli (*χ*^2^ = 2, *df* = 1, *p* = 1; Fig. 4a). As seen for *A. cerana*, when trained to pale yellow (*n* = 20), *T. iridipennis* responded in the tests with a clear preference for yellow (*N* = 344 choices; *ss*_*t*_ = 239.20, *p* < 0.01) and long-wavelength stimuli (*χ*^2^ = 22.57, *df* = 1, *p* < 0.001;Figs. 3b and 4b).

The choices made by colour-naive bumblebees (*B. terrestris*, *n* = 22) were significantly different from random (*N* = 384 choices; *ss*_*t*_ = 82.00, *p* < 0.01; Fig. 3a), with the highest number of approaches to bluish-green. There were significant differences when comparing approaches to short- or long-wavelength stimuli with long-wavelength stimuli receiving more choices (*χ*^2^ = 4.27, *df* = 1, *p* < 0.05; Fig. 4a). Bumblebees trained to pale yellow (*n* = 16) displayed a non-random distribution of choices with a peak for the yellow stimulus (*N* = 242 choices; *ss*_*t*_ = 102.46, *p* < 0.01; Fig. 3b) and generalised preferences towards long-wavelength colours (*χ*^2^ = 13.82,*df* = 1, *p* < 0.001; Fig. 4b).

### Enclosed *A. cerana* foragers

The number of approaches made to the test colours by *A. cerana* foragers enclosed for 2 (*n* = 15), 6 (*n* = 15), and 8 weeks (*n* = 11) were significantly different from expected frequencies (*2 weeks*: *N* = 331 choices; *ss*_*t*_ = 144.79, *p* < 0.01; *6 weeks*: *N* = 219 choices; *ss*_*t*_ = 64.79, *p* < 0.01; *8 week*s: *N* = 212 choices; *ss*_*t*_ = 170.91, *p* < 0.01; Fig. 3c). After training to the achromatic UV-grey stimulus, the enclosed foragers preferred short-wavelength colours (2 weeks: *χ*^2^ = 4.27, *df* = 1, *p* < 0.05; 6 weeks: *χ*^2^ = 12.33, *df* = 1, *p* < 0.001; 8 weeks: *χ*^2^ = 74.27, *df* = 1, *p* < 0.05; Fig. 4c). A large proportion of approaches were directed towards the cyan test stimulus in the 2- and 6-week enclosed foragers. However, there was a shift in the peak of choices from cyan to bluish-green in the 8-week enclosed foragers.

The choice of test stimuli of the 9-week enclosed foragers trained to chromatic pale yellow (*n* = 10) was not different from the expected frequency (*N* = 114 choices; *ss*_*t*_ = 16.75, *p* > 0.05; Fig. 3b), and there were no significant differences in the approaches towards stimuli categorised as short and long wavelength (*χ*^2^ = 0.68, *df* = 1, *p* > 0.05; Fig. 4b). However, these bees seem to have learned, because their responses differed from those of the other three enclosed groups.

## Discussion

Several studies have described spontaneous colour preferences in bees (Lunau 1990; Giurfa et al. 1995; Chittka et al. 2004; Dyer et al. 2016), butterflies (Ilse 1928; Swihart and Swihart 1970; Weiss 1997; Kinoshita et al. 1999; Kandori et al. 2009; Blackiston et al. 2011), moths (Kelber 1996; Goyret et al. 2008), and hoverflies (Lunau and Wacht 1994). The aims of the present study were to explore a comparative approach with bees and to identify potential differences in spontaneous colour responses between temperate and tropical bees, comparing the responses of three social bee species that were tested under the same conditions. The tropical *Apis cerana* and temperate *Bombus terrestris* displayed similar spontaneous colour preferences, whilst the tropical stingless bee *Tetragonula* (*Trigona*) *irridipennis* differed from both of them in their preference curve.

The diversity of methods and stimuli employed in the various studies makes it difficult to compare such preferences across pollinator groups in order to understand their adaptive value and to elucidate the mechanisms underlying them. For instance, the number of colours offered in an experiment or test can range from as little as just two (e.g. Raine and Chittka 2007) to eight, as in our study, to as many as 10 or 12 (Lunau 1990; e.g. Giurfa et al. 1995; Kandori et al. 2009). There are also variations in the spatial arrangements, e.g. colours being displayed once as single stimuli or repeatedly in a testing arena. Whilst many studies present stimuli as flat coloured discs (or as other flat shapes), usually without scent (but see Giurfa et al. 1995; Yoshida et al. 2015), others add stalks or other flower-like elements to provide shapes that extend in all three dimensions.

Also, the manipulative control of colour experience should not detrimentally affect the insect’s development or ability to execute natural behaviours, and sufficient numbers of naive individuals that are mature enough or motivated to forage have to be reared. In the case of bees, naive foragers do not readily display attempts to explore coloured stimuli and have to therefore be pre-trained on neutral (achromatic) stimuli (Giurfa et al. 1995; Chittka et al. 2004; Rodriguez et al. 2004; Orbán and Plowright 2014). Solitary insects without food stores are typically starved after eclosure, for example for up to 2 days in the monarch butterfly (e.g. Blackiston et al. 2011). Finally, the brightness and colouration of the background on which stimuli are presented also plays a role, as it influences the adaptation state of receptors and the colour vision system, but can also potentially provide cues that could change the internal state of some insects, like those that use foliage as oviposition sites. The role of background brightness and colouration for colour detection and spontaneous colour choices has so far been explored in more depth in moths and butterflies than in bees and should be considered when comparing across species (Neumeyer 1980; Kelber 1997; Hempel de Ibarra et al. 2000; Niggebrügge and Hempel de Ibarra 2003; Kinoshita et al. 2008; Kinoshita et al. 2012).

We found in our experiments that bees explored all colours and displayed overall preferences for colours dominated by either long or short wavelengths, rather than showing sharp preferences for a single-colour stimulus. This can be expected when presenting a larger number of colours, as colour generalisation and exploration of non-preferred colours is likely to occur. The number of colours offered and foraging experience, even without colour learning, could affect the outcomes of the test, and first experimental evidence has recently been provided. Blackiston et al. (2011) reported that spontaneous colour preferences in the monarch butterfly *Danaus plexippus* were influenced by the combination of coloured stimuli presented during the tests. Orange was preferred in a six-colour array with yellow being chosen roughly equally to the red and blue stimuli. When however the latter three colours were tested separately, the butterflies showed a very strong preference for the yellow stimulus. This obviously makes it difficult to identify which or whether at all any spontaneous preferences are truly innate.

Our findings differ from those of an earlier study in the Western honeybee (*Apis mellifera*) where 12 colours were displayed and the choice curve was observed to sharply peak for a single stimuli (Giurfa et al. 1995). It is unlikely that species differences would account for this, but it seems plausible that variations in experimental procedures may cause differences in the bees’ choices. Giurfa and colleagues used a scented enclosure when presenting the stimuli, whilst in our study, stimuli were unscented and the bees were not prevented from flying up and away from the test disc. In addition, ‘late’ tests were conducted, testing the bees for a second time after further training trials with neutral stimuli (Giurfa et al. 1995). The preference curves in these second (‘late’) tests were broader, although still displaying somewhat higher preferences for short-wavelength colours. Interestingly, in the ‘late’ tests, bees increased their choices for long-wavelength colours. It could well be that extending the experience of the insect with a rewarding patch, even if it does not contain colourful stimuli increases the willingness of the animals to inspect more colours in a test.

We did not attempt to test further cues in addition to colour that have been reported to modify spontaneous responses in insects, such as odour (Yoshida et al. 2015) or patterns (Lunau 1990; Kelber 1997; Gumbert 2000). It is worth highlighting that we trained and tested bees individually, which has the advantage that interferences from other bees are avoided and the experience of the bees controlled better. Several studies have previously applied training or testing procedures, or both, for groups of bees (Lunau 1990; Giurfa et al. 1995; e.g. Dyer et al. 2016), which could potentially also influence an individual’s choices or the accuracy of recording them.

Bees in our experiments did not show strong preferences for blue and violet colours found in other studies with the same species of bumblebees, *Bombus terrestris* (Lunau 1990; Gumbert 2000; Chittka et al. 2004; Raine and Chittka 2007), and with the Western honeybee, *Apis mellifera* (Giurfa et al. 1995). We argue that spontaneous colour preferences, were they indeed expressions of adaptive innate colour preferences, should be demonstrable under a range of experimental conditions, and we should have seen them in our experiments too. If we perhaps neglected to display a suitably blue colour in our array, we would still expect bees to show generalisation towards short-wavelength colours, preferring those over long-wavelength stimuli. However, this was not the case. We found that colour-naive *Apis* and *Bombus* foragers did not inspect the short-wavelength stimuli more. Proportions of choices were highest to bluish-green in naive *Bombus* and bluish-green and lime-yellow in naive *Apis*, and there was a significant bias towards the long wavelengths. We conclude that the spontaneous preferences are similar between the temperate and the tropical bee species, which does not support the general idea that pollinators with similar visual systems evolved innate colour preferences as an adapative strategy to maximise recognition and exploitation of varied sets of colour cues in flowers across different macroecological regions. Alternatively, it could also be that flowers are too diverse in colour always and anywhere, as they signal to a diversity of pollinators that are mostly generalist foragers (e.g. Schiestl and Johnson 2013). Comparisons of flower colours collected broadly in different regions of the world suggest that this could be the case (Menzel and Shmida 1993; Chittka et al. 1994; Vorobyev and Menzel 1999; Shrestha et al. 2014). It might be therefore necessary to continue advancing studies that look at smaller biogeographical areas to understand how flower cues relate to conditions in specific habitats and how they influence foraging decisions in specific plant-pollinator systems and networks, considering habitat structures, phenology of co-flowering species, degree of foraging specialisation, but importantly also the rewards available within a network of pollinators that share common plant resources.

From this perspective, it is interesting that we observed differences amongst the two tropical bee species which suggests a possible role of innate colour preferences for resource partitioning. The stingless bees showed a different preference curve, more frequently choosing the bluish-green and violet stimuli and less frequently lime-yellow and yellow. Given the long enclosure time of the stingless bees, it is very unlikely that *Tetragonula* foragers were not naive. However, the alternative, less likely possibility would be that we tested old foragers whose choices might have been affected by the long period of enclosure. A comparison with the preference curve of the enclosed *Apis* foragers, however, does not show a full overlap of their choice curves. Thus, it could well be that there are species-specific differences amongst distantly related bee species. It will be worthwhile to explore this further examining colour vision and learning in this species and other stingless bees (e.g. Spaethe et al. 2014).

For Western honeybees *Apis mellifera* and *Bombus* species, it has been extensively shown that conditioned colours determine the subsequent colour choices in foraging bees, even if the rewards fluctuate strongly (Heinrich et al. 1977). These choices are underpinned by life-long memories (Menzel 1967; Menzel 1968; Menzel 1969). Therefore, enclosing experienced *A. cerana* foragers for some time in a flight net appeared to be a suitable solution for extinguishing their colour memories. However, we found that after 2 weeks of enclosure, the bees did not respond in the same way as the naive animals, which suggests that the enclosed bees were not naive but old foragers. After 6 weeks of enclosure, the responses changed; however, overall bees still preferred short-wavelength colours, whereas naive bees had no preference between short- and long-wavelength stimuli. We could not identify the causes for this change, e.g. whether experienced foragers changed their preferences at older age or as a result of colour deprivation, or whether a turnover from experienced to naive foragers was taking place, or indeed a combination of both.

Bees learn diverse colours easily (Menzel 1967; Menzel 1979; Vorobyev et al. 1999; Niggebrügge et al. 2009), although strong colour contrast seems to render coloured stimuli more salient for bees (Niggebrügge and Hempel de Ibarra 2003). It was sufficient to briefly reward bees of each species on pale yellow stimuli with a weak, but perceivable colour contrast, in order to shift their preference curve, as predicted, towards yellow. It has previously been shown that colour experience overrides the expression of spontaneous preferences in bees. Giurfa et al. (1995) found that a single pre-training trial with a strongly contrasting colour is sufficient to affect colour preferences of bees in a subsequent test. Here, we extend those findings and suggest that even with less salient colours, learning is sufficiently fast and robust to change spontaneous colour preferences.

Most pollinators are generalists and deal with unpredictable and changing spatial distributions, abundances, and levels of rewards of flowers by readily learning their signals, colours, and odours. Future work should continue to uncover the functional links between spontaneous preferences, learning processes, and decision-making.

## Electronic supplementary material

Fig. S1 (PDF 82 kb)
