# Supplementary material for: A comparative analysis of colour preferences in temperate and tropical social bees
Source: Naturwissenschaften. 2018 Jan 2;105(1):8. doi: 10.1007/s00114-017-1531-z (PMC5750331; doi:10.1007/s00114-017-1531-z)
Supplement: Supplementary file 1 — (PDF 82 kb) [file 114_2017_1531_MOESM1_ESM.pdf]

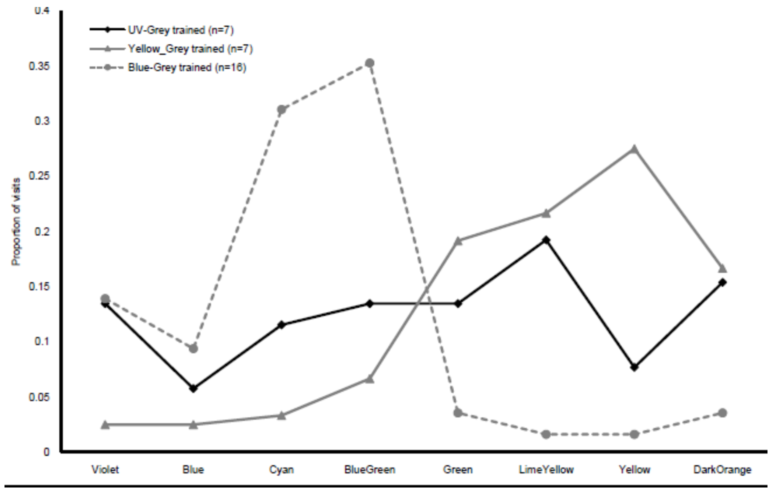

Fig S1. Pilot experiments with free-flying *A. cerana* foragers that were experienced with natural flowers. Bees generalized towards short wavelength colours after training with Pale-Blue stimuli (*sst* = 758.99, *p*=0.0002, $\chi^2_1 = 59.29$, *df* = 1, $p < 0.001$) and long wavelength colours (*sst* = 141.71, *p*=0.0062, $\chi^2_1 = 47.61$, *df* = 1, $p < 0.001$) after training with a Pale-Yellow stimuli. After training to UV-Grey stimuli, bees did not exhibit any preference patterns (*sst* = 4.85, *p*=0.860,$\chi^2_1 = 1.21$, *df* = 1, $p = 0.27$), which is likely to be an effect of mixed previous experiences with flower colours that the foragers had.
